# Dr. Magennis's Remarks and Observations on Digitalis

**Published:** 1800-02

**Authors:** James Magennis

**Affiliations:** Norman Cross


### *To the Editors of the Medical and Phyſical Journal.*

Gentlemen,

EVERY man converſant in the practice of phyſic, and who reflects with due conſideration on the importance of the ſubject, muſt perceive with pleaſure the many benefits which are likely to reſult to the community in general, and to the medical profeſſion in particular, through the medium of your excellent Miſcellany. To the remote country practitioner, cut off from a knowledge of the more recent improvements and diſcoveries of of the capital, it will prove an acquifition of the higheft importance; it not only conveys a valuable ftock of information to all, but likewife ferves to ftimulate the indolent, and awaken a fpirit of refearch in the more learned part of the profeffion. The truth of this obfervation has been already evinced in the numerous experiments inftituted by different gentlemen, in order to afcertain with precifion the medicinal powers of various active remedies. Among thefe, the Digitalis purpurea, has defervedly attracted a large fhare of the public attention; and at this we are not to be furprifed, when it is confidered, that a belief began to prevail of its poffeffing peculiar fpecific powers in the cure of phthifis pulmonalis; a difeafe which had, hitherto, but too often baffled the fkill of the ableft phyficians, from the days of Hippocrates to the prefent time, and which has proved particularly deftructive to the inhabitants of this ifland. Happy, indeed, will it be for a large portion of the inhabitants of Europe, fhould future experience and inquiry confirm the high encomiums with which it has been of late fo much honoured. The favourable reports circulated of its anti-phthifical effects, from various quarters of high refpectability, induced me to give it a trial; and it is a duty incumbent on every profeffional man, to add his little ftock of information to the general mafs of knowledge, as well as to contribute his mite towards elucidating a queftion, on the folution of which the lives and happinefs of many of his fellow creatures depend.

If the following cafe and obfervations be deemed worthy of infertion in your valuable publication, they are much at your fervice. The only merit it claims, is confined to accuracy, and the moft religious adherence to truth. The pulfe was examined fyftematically, morning, afternoon, and night, except the two firft days, under my own care, or that of Mr. Woodham, head-affiftant, a gentleman of great diligence and ability. It was deemed neceffary to be thus particular, from the extraordinary changes which invariably marked the daily ftate of the pulfe, efpecially after the patient came to be completely under the influence of the medicine, and from an anxious defire of becoming acquainted with the exact properties of the digitalis.

It was exhibited regularly three times a day, at the hours of 8, A. M. 1 and 7, P. M. The periods chofen to examine the pulfe were, 10, A. M. 5 and 9, P. M. which, to prevent prolixity and repetitions, need not be again mentioned.

I remain, with great refpect, GENTLEMEN,

Your very humble fervant,

JAMES MAGENNIS, M. D.

*Norman Crofs, Dec.* 27, 1799.

 The

The ſubject of the following caſe, Bertrand Stroed, a Dutch priſoner, is a young man of 27 years of age, thin viſage, long neck, high ſhoulders, and pale complexion: he was admitted into the hoſpital on the 29th of December, 1798, labouring under ſevere pneumonic affections, which, after ſome time, terminated in a troubleſome diarrhœa; he was, however, diſcharged on the 21ſt of Feb. 1799, pretty well recovered, and continued in the priſon till the 9th of Auguſt following, when he was again received into the hoſpital. It ought to have been premiſed, that he had been affected with cough and pain of the ſternum, for two months previous to his arrival at this priſon; that he was very unwell for ſeveral weeks before the ſecond admiſſion, although he made no application, and that the cough continued more or leſs all the time. I found him much emaciated, with a very troubleſome, irritating cough; pain about the ſcrobiculus cordis; difficult and oppreſſed reſpiration, eſpecially when he attempted to lie on either ſide; expectoration profuſe; pulſe from 110 to 120; hard but not full; a dry, hot ſkin, and at no time ſubject to nightly perſpirations. He was treated with various remedies, chiefly on the antiphlogiſtic plan, till the 29th, without any change for the better, except the reduction of the pulſe to 100; but all the other ſymptoms were progreſſively worſe;—the expectoration had increaſed, being now, as in fact it had been for ſome time paſt, evidently purulent, with a ſmall circular, rouge-like, appearance on each cheek. His appetite, however, continued tolerable, diet compoſed principally of milk with a little animal food, and as the debility was extreme, he was allowed four glaſſes of wine daily, immediately on commencing the digitalis, which he did on this day the 29th, in the following draught: ℞. Kali p$^{t}$. ℈j ſuc. limon. ℥ ſs. aq. puræ ʒj tinct. digit. g$^{t}$. x. ter in die ſumend. The pulſe at 10, A. M. 96, at 4, P. M. 88. The 30th, Tinct. g$^{t}$. xv. p. 100, 68. The 31ſt, p. 92, 98, 70. Sept. the 1ſt, Tinct. g$^{t}$. xxx. p. 90, 98, 70. The 2d, Tinct. g$^{t}$. XL. p. 95, 98, 60. The 3d, p. 80, 72, 64. The 4th, Tinct. g$^{t}$. L. p. 90, 78, 64, general ſymptoms in every reſpect the ſame. The 5th, p. 88, 64. The 6th, Tinct. g$^{t}$. LX. p. 80, 64, 52. The 7th, p. 84, 72, 60. The 8th, p. 88, 80, 64. The 9th, Tinct. g$^{t}$. LXX. p. 96, 84, 66. The 10th, 78, 60, 56. The 11th, p. 92, 84, 50. The 12th, p. 96, 72, 50; pulſe intermits; vertigo with nauſea, and vomitted in the morning. The 13th, p. 80, 72, 46, intermiſſions continue, with vertigo and nauſea; vomitted again in the morning; the firſt doſe omitted. The 14th, as the dizzineſs, nauſea, and other diſagreeable ſenſations ſtill continue, the doſe was reduced to Tinct. dig. g$^{t}$. xxx. in aq. menth. ℥ſs. p. 92, 74, 50. The 15th, nauſea, vertigo,

&c.

&c. removed. Tinct. g^r. xl. p. 104, 76, 42. The 16th, Tinct. g^t. xlv. p. 72, 72, 52, slight dizziness, the appetite good, body regular. The 17th, Tinct. g^t. l. p. 90, 68, 48. The 18th, Tinct. g^t. lv. p. 78, 60, 46. The 19th, Tinct. g^t. lx. p. 80, 72, 44. The 20th, Tinct. g^t. lxv. p. 90, 6[illegible], 38, expectoration the same, head free. The 21st, vomitted the morning dose, p. 96, 60, 42. The 22d, again vomitted, in consequence of which the other two doses were omitted. P. 92, 68, 44, intermitted sometimes every third, fifth, and seventh pulsation; and sometimes two pulsations were lost. The 23d, the dose as on the 20th. P. 60, 52, 34, intermitted during morning, but regular in the evening. The 24th, no alteration in the expectoration, p. 60, 48, 44. The 25th, p. 86, 84, 40. The 26th, p. 48, 90, 40, intermissions as on the 22d. The 27th, p. 72, 80, 44, irregular. The 28th, p. 48, 72, 36. The 29th, p. 60, 80, 40. The 30th, p. 60, 80, 40, small and scarcely perceptible. Oct. the 1st, p. 86, 50, 36, vomitted morning and evening dose. The 2d, Tinct. g^t. l. p. 56, 66, 36, intermitted the whole day, but no nausea. The 3d, p. 52, 88, 38, very regular all the afternoon. The 4th, p. 50, 50, 38, intermitted, vomitted the morning dose. The 5th, p. 60, 84, 40, again vomitted morning dose. The 6th, p. 46, 80, 40, slight nausea with vertigo. The 7th, p. 60, 74, 40, vertigo continues. The 8th, p. 80, 50, 36, dizziness the same. The 9th, p. 68, 90, 38, expectoration visibly diminished. The 10th, p. 48, 80, 48, weak and irregular, but less dizzy than on the two preceding days. The 11th, p. 72, 60, 36. The 12th, p. 84, 90, vertigo gone, expectoration diminishing. The 13th, p. 60, 52, 38, full with intermissions. The 14th, p. 48, 100, 46. The 15th, p. 44, 71, 39. The 16th, p. 44, 72, 44, weak and irregular, expectoration much diminished. The 17th, p. 52, 80, 36. The 18th, p. 40, 72, 34, vomitted the morning dose. The 19th, Tinct. g^t. xl. p. 44, 50, 40, intermissions, vomitted morning dose, in the evening regular and full. The 20th, p. 56, 62, 40, expectoration diminished to one half, cough much better, pain of the scrob. cord. greatly relieved. The 21st, p. 44, 94, 44, regular without nausea. The 22d, p. 45, regular and full; 60, irregular; 40, regular without dizziness. The 23d, p. 56, 100, 44, intermitted. The 24th, p. 44, 80, 40. The 25th, Tinct. g^t. l. p. 60, 120, 40, expectoration still farther diminished. The 26th, p. 48, 40, 40. The 27th, p. 80, 70, 38, expectoration about 7 oz. The 28th, p. 60, 88, 40. The 29th, p. 60, 88, 38. The 30th, p. 52, 60, 44, intermissions with vertigo and vomittings. The 31st, the tinct. reduced to g^t. xxx, p. 60, 52, 40. Nov. 1st, Tinct. g^t. xxxv, p. 64,

 50,

50, 38, the expectoration under three ounces, cough gone except in the night time, pain entirely removed. The 2d, p. 52, 84, 40. The 3d, Tinct. g^t. XL, p. 84, 80, 42, complained of oppreſſion on lying down. The 4th, p. 76, 64, 44. The 5th, p. 62, 42, reſpiration better. The 6th, p. 44, 100, 38. The 7th, p. 94, 90, 36. The 8th, p. 40, 80, 40, expectoration three ſpoonfuls only. The 9th, p. 68, 42, 38, an extremely debilitated pulſe. The 10th, p. 48, 50, 42. The 11th, p. 52, 70, 46. The 12th, p. 48, 50, 46, weak with irregular intermiſſions. The 13th, Tinct. g^t. LV, p. 60, 52, 40, irregular but full. The 14th, p. 68, 70, 50, irregular all day, the cough again troubleſome with dyſpnœa; the cough previous to this was confined to a little on firſt lying down. The 15th, emp. canth. ſcrob. cord. p. 52, 64, 46, the breathing relieved. The 16th, p. 52, 64, 46, vomitted the morning doſe. The 17th, Tinct. g^t. XXX, tinct. op. camph. ʒj. p. 52, 60, 40, irregular cough, and dyſpnœa troubleſome. The 18th, p. 100, 60, 46, expectoration increaſed, but had no diſagreeable fætor, and was free from purulency. The 19th, p. 54, 72, 38, cough better, expectoration diminiſhed. The 20th, Tinct. g . XL. p. 60, ſmall and irregular; 46, 38, full and regular. The 21ſt. p. 50, 40, 40. The 22d, Tinct. g^t. L. p. 48, 70, 38, very weak and ſmall. The 23d, p. 44, 44, 40, intermit. complains of pain and weakneſs in the right arm. The 24th, p. 52, 50, 42, expectoration pure mucus, all appearance of purulence gone for ſome days. The 25th, p. 52, 98, 39. The 26th, p. 76, 86, 40, pain and weakneſs of the arm continue; nothing to be perceived; great oppreſſion with dyſpnœa in an horizontal poſture. The 27th, p. 100, 52, 46, dyſpnœa notwithſtanding a bliſter, inter ſcap. yeſterday. The 28th, p. 68, 64, 44, irregular in the morning, more ſteady in the evening, appetite on the decline. The 29th, p. 52, 62, 40. The 30th, p. 44, 64, 40. December 1ſt, p. 52, 73, 40. The 2d, p. 64, 100, 40. The 3d, p. at 4 P. M. 64 at 9, P. M. 32, weak but regular, and notwithſtanding ſuch ſurpriſing reduction, he is free from nauſea, vertigo, and in other reſpects the ſame as yeſterday. During the 4th, 5th, and 6th, the ſtate of the pulſe, owing to accident, omitted to be put down; but the digit. continued the ſame. The 7th, Tinct. g^t. LX, p 42, 92, 38. The 8th, p. 60, 80, 48, expectoration and dyſpnœa the ſame, pain and weakneſs of the arm removed. The 9th, Tinct. g^t. LXV, p. 57, 72, 40. The 10th, p. 52, 50, 40. The 11th, p. 52, 40, 36. The 12th, p. 92, 42, 40, ſlight nauſea this morning. The 13th, p. 80, 82, 60, the expectoration again diminiſhed to 3 oz. The 14th, p. 52, 50, 46, ſmall and irregular in the morning, but the contrary in the evening, the expectoration expectoration two table fpoonfuls and entirely free from puru-lency. The 15th, the weather about this time fet in extremely fevere, and he had in confequence a frefh attack; the cough, dyfpnoea, and expectoration became more troublefome and pro-fufe, which continued for feveral days; but all thefe bad fymp-toms have again difappeared, and he now appears to advance with a firm pace towards a perfect cure, (the 26th,) although the cold continues unabated. He has been all along extremely fufceptible of being affected by changes in the atmofphere, and unfortunately this has been too much the cafe during the whole year, efpecially fince the commencement of winter. I am inclined to impute the protraction of his cure to local cir-cumftances conftantly operating, of which the principal were, long continued eafterly winds, blowing over a deep marfhy country, to which the fcite of the prifon is much expofed, and the common effects of imprifonment on a mind already debili-tated by a confuming difeafe. The power of the medicine was moft confpicuous in diminifhing the quantity, changing the quality, and removing the fætor of the expectoration, as well as in leffening the force of the vafcular fyftem.

If we attentively confider the hiftory of all the cafes hitherto publifhed by different authors, refpecting the peculiar effects of the digitalis, nothing fatisfactorily decifive can be inferred, if we except the two cafes of Maris and Grimes, fo accurately related by the ingenious Dr. Drake, of Hadleigh; but even in thefe, the ftate of the pulfe was only attended to once a day, and, from the hiftory already narrated, it muft be manifeft that the action of the medicine on the circulation, can only be ac-curately defined by a more frequent daily examination of the pulfe, &c.

The cafes enumerated by Dr. Fowler, and inferted, like the former, in Dr. Beddoe's valuable volume of Contributions, are fo vague and defultory, that nothing more than very indefinite general refults can be collected; as the mode of preparing the medicine, dofe, &c. as well as its particular effects on the head, ftomach, and pulfe, are related very defectively. To thofe re-cited by Dr. Bree, though more methodical than the preceding, I muft object for nearly fimilar reafons; it would appear like-wife that the fubjects on whom he tried the experiments were, what in medical language is generally termed, loft cafes; in fact, the Doctor fays fo himfelf, in very explicit words. Page 432, his obfervations are, "the remaining fix cafes were too far ad-vanced to have been capable of cure from any medicine." In the three cafes which he terms incipient, one of them only per-fevered in taking the medicine for any length of time, and it proved permanently fuccefsful: Of the others, Harrifon took the the powder nineteen days, and then difcontinued. Underhill commenced with the tincture on the 8th July; but the quantity he took, or the time he perfevered in its ufe, is not mentioned, only that he purfued it till anorexia and weaknefs made him lofe all patience. It is to be obferved, alfo, that in the greater number, the powder was adminiftered, a form of exhibiting which renders it impoffible to regulate the dofe with that degree of precifion fo neceffary to be attended to, when active remedies are to be employed. From thofe unfuccefsful cafes, then, to draw unfavourable conclufions refpecting the anti-phthifical effects of the digitalis, is moft affuredly unjuft, and what the premifes will by no means warrant.

The remarks of Dr. Maclean, of Sudbury, on the common effects of full dofes of the digitalis, were in general found perfectly juft and conformable to the phænomena obferved in Stroed's cafe; but the Doctor was evidently miftaken when he afferted, page 119, that thofe who expect to fucceed in reducing the pulfe to any confiderable degree, without inducing vertigo, with vomittings, will be difappointed. The preceding cafe proves, that the pulfe, after a certain fpace of time, was, in the evening, almoft conftantly at or under forty, frequently at 38, 36, 34, and once at 32, when neither the head nor ftomach was affected; even at the extraordinary reduction of 32, the pulfe was free from intermiffions, and the patient, on being afked, if he felt any particular uneafy fenfations, replied, not in the leaft. What is ftill more remarkable, is, that the pulfe was always found more regular, and generally fuller, when under 40, than above that and 50.

On the neceffity of having a ftandard formula, in order to regulate general practice, I fully agree with Dr. Maclean, and the tincture ufed in the prefent cafe was made from his prefcription;—it was found impoffible to conform to that of Dr. Drake—one ounce of grofs powder abforbed four ounces of proof fpirit entirely; and I am perfuaded his laft improvement of one ounce to five will be found likewife inconvenient. A tincture prepared according to Dr. Maclean's method, may be adminiftered with more freedom, and the dofe better regulated.

The digitalis fhewed no tendency whatever to act as a diuretic, nor were the inteftines at all affected by it, the body having continued perfectly regular from the commencement to the end.

Upon the whole, then, after maturely confidering the phænomena attending the internal ufe of the digitalis in the prefent cafe, and comparing them with thofe obferved by other gentlemen, we fhall be warranted in drawing the following general conclufions.

*First*

*First*, That this medicine, in the form of tincture, if carefully and gradually exhibited, may be ultimately given in large doses, and continued for months with the most perfect safety to the patient.

*Secondly*, That after a certain length of time, when the system is charged, and completely under its influence, it possesses a power of arresting the motion of the heart and arteries in a surprising manner; but that, under such influence, they are exquisitely susceptible of being roused into action on the slightest bodily exertion.

*Thirdly*, That the pulse may be brought to a degree of reduction hitherto thought incompatible with the existence of animal life, for any considerable time, without danger to, or even any material derangement of, the animal œconomy.

And *Lastly*, That the criterion by which we are to be guided in regulating the increase, diminution, or total omission of the medicine, depends on the phænomena arising from its action on the sensorium and stomach, and not on the quantum of reduction in the pulse.

